# Effect of nonsurgical periodontal treatment in conjunction with either systemic administration of amoxicillin and metronidazole or additional photodynamic therapy on the concentration of matrix metalloproteinases 8 and 9 in gingival crevicular fluid in patients with aggressive periodontitis

**DOI:** 10.1186/s12903-015-0048-0

**Published:** 2015-05-26

**Authors:** Anna Skurska, Ewa Dolinska, Małgorzata Pietruska, Jan K. Pietruski, Violetta Dymicka, Halina Kemona, Nicole B. Arweiler, Robert Milewski, Anton Sculean

**Affiliations:** Department of Periodontal and Oral Mucosa Diseases, Medical University of Białystok, ul. Waszyngtona 13, 15-269 Białystok, Poland; Private Practice, ul. Waszyngtona 1/34, 15-269 Białystok, Poland; Department of Clinical Laboratory Diagnostics, Medical University of Białystok, ul. Waszyngtona 15, 15-269 Białystok, Poland; Department of Periodontology, Philipps-University of Marburg, Georg-Voigt-Str. 3, D-35039 Marburg, Germany; Department of Statistics and Medical Informatics, Medical University of Białystok, ul. Szpitalna 37, 15-295 Białystok, Poland; Department of Periodontology, Dental School University of Bern, Freiburgstrasse 7, 3010 Bern, Switzerland

**Keywords:** Nonsurgical periodontal therapy, Photodynamic therapy, Systemic antibiotics, Matrix metalloproteinases, Aggressive periodontitis

## Abstract

**Background:**

To evaluate in patients with aggressive periodontitis (AgP) the effect of nonsurgical periodontal treatment in conjunction with either additional administration of systemic antibiotics (AB) or application of photodynamic therapy (PDT) on the gingival crevicular fluid (GCF) concentration of matrix metalloproteinases 8 and 9 (MMP-8 and -9).

**Methods:**

Thirty-six patients with AgP were included in the study. Patients were randomly assigned to treatment with either scaling and root planing (SRP) followed by systemic administration of AB (e.g. Amoxicillin + Metronidazole) or SRP + PDT. The analysis of MMP-8 and -9 GCF concentrations was performed at baseline and at 3 and 6 months after treatment. Nonparametric U-Mann-Whitney test was used for comparison between groups. Changes from baseline to 3 and 6 months were analyzed with the Friedman’s ANOVA test with Kendall’s index of consistency.

**Results:**

In the AB group, patients showed a statistically significant (*p = 0.01*) decrease of MMP-8 GCF level at both 3 and 6 months post treatment. In the PDT group, the change of MMP-8 GCF level was not statistically significant. Both groups showed at 3 and 6 months a decrease in MMP-9 levels. However, this change did not reach statistical significance.

**Conclusions:**

Within the limits of the present study, it may be suggested that in patients with AgP, nonsurgical periodontal therapy in conjunction with adjunctive systemic administration of amoxicilin and metronidazole is more effective in reducing GCF MMP-8 levels compared to the adjunctive use of PDT.

## Background

Aggressive periodontitis (AgP) is characterized by a rapid destruction of periodontal supporting tissues leading to pocket formation, loss of clinical attachment and alveolar bone. When left untreated, AgP results in deterioration of tooth prognosis, thus severely affecting long-term tooth survival [1, 2]. The etiology of AgP is complex, consisting of potential local host response deficiencies coupled with infection with certain periodontopathic microorganisms such as *Aggregatibacter actinomycetemcomitans (A.a.) and Porphyromonas gingivalis (P.g.)* [3–5]. Generally, patients with AgP show a more moderate response to mechanical debridement alone [6, 7]. This is probably due to the tissue invading capacity of *A.a.* and *P.g.* [3–5] which makes their elimination by means of mechanical therapy alone extremely difficult [6, 7]. The systemic administration of amoxicillin and metronidazole in conjunction with mechanical debridement (SRP) has been shown to be a successful method to reduce or eliminate *A.a.* and *P.g.* and to improve the clinical outcomes [6–8]. However, the use of antibiotic therapy is often accompanied by undesirable side effects involving the digestive system and genitourinary tract as well as the development of bacterial resistance [9, 10]. Furthermore, patient compliance appears to be also an important issue related to the use of systemic antibiotics, which in turn, may influence the development of bacterial resistance and the clinical outcomes [10–12].

The use of photodynamic therapy (PDT) involving the combination of visible light (e.g. usually a diode laser) and a photosensitizer in conjunction with SRP has been shown to substantially reduce the bacterial load and to result in improved clinical outcomes compared to the use of SRP alone [13, 14]. The results of a very recent controlled clinical study have shown that in patients with AgP, nonsurgical periodontal therapy in conjunction with either systemic administration of amoxicillin and metronidazole or followed by 2 x topical application of aPDT resulted in significant clinical improvements evidenced by probing depth (PD) reduction, gain of clinical attachment (CAL) and reduction of inflammation as evidenced by improvements in bleeding on probing (BOP) scores at 3 and 6 months. However, treatment with amoxicillin and metronidazole, has led to statistically significantly higher reductions in mean PD and in the number of pockets ≥ 7 mm compared with the application of PDT [15, 16].

Gingival crevicular fluid (GCF) is an inflammatory exudate, which increases significantly in inflammatory conditions within periodontal tissues [17–22]. The increase of GCF is accompanied by migration of neutrophils, which leads subsequently to a release of matrix metalloproteinases 8 and 9 (MMP-8 and -9) from their granules [17–22]. Several studies have indicated that extracellular matrix metalloproteinases (MMPs), especially MMP-8 and MMP-9, may play a major role in periodontal tissue destruction [20]. Söder [23] and Leppilahti [24] observed significantly higher levels of MMP-8 and MMP-9 in GCF of patients with periodontal disease compared to healthy controls. High levels of MMP-8 and MMP-9 in GCF were found in patients with chronic and aggressive periodontitis [25, 26]. Therefore, during the last years, it was suggested to use the presence and concentration of certain MMPs, e.g. MMP-8 MMP-8 or MMP-9 GCF levels to monitor periodontal tissue conditions [21–26]. On the other hand, the data evaluating the effect of various periodontal treatment protocols on the MMP levels in the crevicular fluid are still limited data. Moreover, to the best of our knowledge, at present it is unknown to what extent nonsurgical periodontal therapy in conjunction with either additional administration of systemic antibiotics (AB) or application of PDT may influence MMP-8 or MMP-9 GCF levels in patients with AgP.

Therefore, the aim of this study was to evaluate the effect of nonsurgical periodontal treatment in conjunction with either additional administration of amoxicillin and metronidazole or application of PDT on the concen tration of MMP-8 and -9 in GCF.

## Methods

### Patients and study design

The study design, patient population, treatment protocol together with the 3 and 6 months results have been described previously in great detail [15, 16]. Briefly, the study was an examiner-blind, prospective, randomised, intra-individual comparative, single-centre clinical study. Prior to participation, the purpose and risks of the investigation were fully explained to all participants and written informed consent was obtained from all patients. The study protocol was approved by the ethical committee of the Bialystok University (approval no. R-I-002/307/2009) and conducted according to the principles outlined in the Declaration of Helsinki on experimentation involving human subjects in the Department of Periodontology, Medical Academy Bialystok, Poland.

Thirty-six patients (24 females, 12 males) suffering from aggressive periodontitis [1] with at least 3 sites with PD ≥ 6 mm were recruited for the study and randomized in two parallel groups of 18 patients each.

Following one or more appointments including thorough oral hygiene instructions and supragingival scaling and polishing, all pockets ≥ 4 mm were treated by means of SRP using ultrasonic scalers and hand instruments. All treatments were performed under local anaesthesia. In the aPDT group, the photosensitizer (HELBO® Blue Photosensitizer, Helbo Photodynamic Systems GmbH & Co KG; Wels, Austria) was applied into the pockets from apically to coronally. After 3 min. the pockets were rinsed with sterile NaCl solution and subsequently irradiated with a diode laser tip (HELBO® minilaser 2075Fdent, Helbo Photodynamic Systems GmbH & Co KG; wavelength 660 nm) for one minute.

In the antibiotic group (AB), the patients were prescribed 375 mg of amoxicillin and 250 mg of metronidazole 3 × daily for 7 days, starting on the day of SRP [7, 15, 16].

### GCF sampling

GCF was obtained from the deepest periodontal pocket per patient (≥6 mm deep), chosen at baseline. After isolation of the tooth with cotton rolls, dental plaque was gently removed and the tooth was air-dried. GCF was collected with sterile paper strips (Periopaper, Interstate Drug Exchange, Amityville, NY, USA), introduced into the periodontal pocket at the depth of 1-2 mm for 30s. The GCF volume (Sulcus Fluid Flow Rate- SFFR) absorbed in a paper strip was measured with a calibrated device (Periotron 8000, Oraflow, Plainview, NY). Following measurement, the samples were immediately placed in Eppendorf tubes containing 20 μl PBS (phosphate buffered saline) and frozen at − 20 °C. At 3 and at 6 months GCF was collected exactly from the same sites as at baseline.

### GCF MMP-8 and MMP-9 analyses

The MMP-8 and -9 GCF concentrations were evaluated with the ELISA method using commercially available kits (R&D Systems, Minneapolis, MN, USA) according to the producer’s manual.

### Statistical analysis

Statistical analysis was perfomed with Statistica 10 software (StatSoft, Tulsa OH, USA). Nonparametric U-Mann-Whitney test was used for comparison between groups. For the statistical evaluation of the changes from baseline to 3 and 6 months the Friedman’s ANOVA test with Kendall’s index of consistency was used. The results were considered statistically significant at *p* < 0.05.

## Results

Prior to treatment, the levels of SFFR, MMP-8 and MMP-9 in GCF did not show statistically significant differences between the two groups. SFFR values diminished significantly (*p = 0.006* A; *p = 0.001* PDT) in both groups, but there were no differences between the groups at 3 and 6 months (Table 1).

**Table 1:** Comparison of the mean values of SFFR (± standard deviation) between groups and between examinations

Examination	A	PDT	Group versus group; p
I	121.15 ± 37.42	128.55 ± 38.48	0.51 NS
II	81.10 ± 50.99	91.20 ± 43.16	0.30 NS
III	89.85 ± 45.24	96.30 ± 43.10	0.61 NS
Timing;	0.006*	0.001*	

A—patients with aggressive periodontitis, who underwent SRP and antibiotic therapy

PDT-patients with aggressive periodontitis, who underwent SRP and PDT

I—baseline

II—3 months after treatment

III—6 months after treatment

*NS* not significant

*p < 0.05; statistically significant

In the AB group, patients showed a statistically significant (*p = 0.01*) decrease of MMP-8 GCF level at both 3 and 6 months post treatment. In the PDT group, a decrease of MMP-8 GCF level was also noted, but the change was not statistically significant.

At 3 and 6 months statistically significant differences were found between the antibiotic and PDT groups (*p = 0.04* and *p = 0.01*, respectively) (Table 2).

**Table 2:** Comparison of the mean values of MMP-8 (± standard deviation) between groups and between examinations

Examination	A	PDT	Group versus group; p
I	42.18 ± 38.19	61.30 ± 63.43	0.62 NS
II	12.03 ± 12.08	35.81 ± 41.94	0.04*
III	13.23 ± 8.71	30.32 ± 29.77	0.01*
Timing;	0.01*	0.06 NS	

A—patients with aggressive periodontitis, who underwent SRP and antibiotic therapy

PDT-patients with aggressive periodontitis, who underwent SRP and PDT

I—baseline

II—3 months after treatment

III—6 months after treatment

*NS* not significant

*p < 0.05; statistically significant

Compared to baseline, the MMP-9 levels showed, in both groups, a decrease at 3 and 6 months. However, this change did not reach statistical significance (Table 3).

**Table 3:** Comparison of the mean values of MMP-9 (± standard deviation) between groups and between examinations

Examination	A	PDT	Group versus group; p
I	172.26 ± 106.65	352.92 ± 73.72	0.35 NS
II	68.36 ± 66.88	199.55 ± 169.39	0.13 NS
III	58.2 ± 33.05	198.42 ± 107.64	0.80 NS
Timing	0.09 NS	0.17 NS	

A—patients with aggressive periodontitis, who underwent SRP and antibiotic therapy

PDT-patients with aggressive periodontitis, who underwent SRP and PDT

I—baseline

II—3 months after treatment

III—6 months after treatment

*NS* not significant

*p < 0.05; statistically significant

## Discussion

The results of the present study revealed that in patients with AgP the use of amoxicillin and metronidazole resulted in a statistically significant reduction of MMP-8 GCF levels at 3 and 6 months after treatment. Treatment with SRP + PDT yielded a reduction in MMP-8 GCF level, but this change was not statistically significant. Compared to the application of PDT, the systemic administration of amoxicillin and metronidazole has led at both evaluation time points to statistically significantly higher reductions of MMP-8 CGF levels compared to the application of PDT. These findings are in line with the clinical results, which have demonstrated statistically and clinically significantly higher clinical improvements in amoxicillin plus metronidazole group compared to the PDT one (i.e. at six months following therapy the total number of pockets ≥ 7 mm was reduced from 141 to 3 in the antibiotic group, while the corresponding values were 137 and 45, respectively in the PDT one) [15, 16]. Comparable findings were also reported by Goncalves et al. [26] who found dramatic decrease in MMP-8 and-9 levels after SRP and systemic use of Amoxicillin + Metronidazole. Thus, the present changes of GCF MMP-8 levels reflect the clinical improvements and provide additional evidence for the efficacy of amoxicillin and metronidazole in the treatment of patients with AgP [6–8, 15, 16].

The fact that the application of PDT in conjunction with SRP has failed to result in statistically significant reduction of MMP-8 CGF level compared to baseline appears to indicate that in patients with AgP, the application of PDT additionally to SRP leads to a more moderate healing response compared to the systemic administration of amoxicillin and metronidazole. These findings might be explained by the capacity of certain periodontopathic bacteria such as *A.a.* and *P.g.* to adhere to cells and to invade cells even in deeper soft tissues layers surrounding periodontally diseased teeth [3].

Since it has been demonstrated that the cytotoxic product which results following PDT, e.g. O_2_, cannot migrate at a deeper distance than 0.02 μm after its formation, it may be anticipated that PDT is more suitable for local application without reaching distant molecules, cells or organs [27]. Hence, it appears that the systemic administration of an adequate dose of specifically selected antibiotics may have a substantially higher potential to reach the cells harboring periodontopathic bacteria than the application of PDT.

On the other hand, it has to be kept in mind that despite the fact that the clinical improvements obtained in the antibiotic group were significantly higher compared to those obtained with PDT, the use of PDT has also led to statistically significant improvements compared to baseline [15, 16]. These results corroborate, at least in part, very recent findings in patients with AgP evaluating the adjunctive use of PDT to SRP. At 90 days, in deep periodontal pockets ≥ 7 mm at baseline, the adjunctive application of PDT yielded statistically significantly higher decrease in PD and clinical attachment gain compared to treatment with SRP. The SRP + PDT group also demonstrated significantly less periodontal pathogens of red and orange complexes and a lower ratio IL-1β/IL-10 than the SRP Group [28]. However, when comparing the aforementioned results to our findings, it has to be kept in mind, that differences in the frequency of PDT application after SRP (i.e. 2× versus 4×) may have also influenced the outcomes. Taken together, the available data appear to suggest a potential role of PDT in modulating periodontal inflammation [13, 14].

In the present study, the MMP-9 levels were reduced in both groups. Despite the fact that the reduction of MMP-9 levels was greater in the antibiotic group, the differences between the examinations periods and among the groups did not reach statistical significance. This finding is somewhat in contradiction with those reported data by Gocalves et al. [26] who have reported statistically significant reductions in MMP-1, MMP-8, MMP-9, MMP-12, and MMP-13 levels up to 6 months, comparable to healthy sites. Significant correlations were noted between MMP-2, MMP-3, MMP-8, MMP-9, MMP-12, and MMP-13 levels and percentage of sites with PD >4 mm. Other authors have also reported a reduction of multiple inflammatory biomarkers including MMP-9 levels after different modalities of periodontal therapy, but the responses were inconsistent among the subjects while the changes of inflammatory markers correlated poorly with clinical parameters [29]. Moreover, GCF MMP-8 and MMP-9 data showed very high standard deviations, which may be related with individual variability. Obviously, these very high standard deviations prevented detection of statistically significant differences between different time points and between the study groups at the same time points.

These inconsistent findings are also in line with the conclusions of a sytematic review which indicated that at present, no single or combination of markers exists that can reveal periodontal destruction adequately and further studies are needed to provide an objective demonstration of the use of such markers to monitor periodontal destruction [22].

When interpreting the present results, it should be mentioned that from a scientific point of view a third group of AgP patients, treated with SRP alone, would have been of interest to evaluate and compare the additive effects of antibiotics and PDT. On the other hand, substantial data from the literature has provided clear evidence indicating that the use of systemic antibiotics represents today’s state of the art in the treatment of AgP [6–8]. Therefore, for obvious ethical reasons, the inclusion of a third treatment arm using SRP alone was not feasible.

## Conclusions

Within their limits, the present results indicate that in patients with AgP, nonsurgical periodontal therapy in conjunction with adjunctive systemic administration of amoxicilin and metronidazole is more effective in reducing GCF MMP-8 levels compared to the adjunctive use of PDT.
